# CD4 and CD8 T Cell Memory Interactions Alter Innate Immunity and Organ Injury in the CLP Sepsis Model

**DOI:** 10.3389/fimmu.2020.563402

**Published:** 2020-11-20

**Authors:** Matthew D. Taylor, Tiago D. Fernandes, Alexander P. Kelly, Mabel N. Abraham, Clifford S. Deutschman

**Affiliations:** ^1^ The Division of Critical Care Medicine, Department of Pediatrics, The Feinstein Institutes for Medical Research, Manhasset, NY, and Cohen Children’s Medical Center/Northwell Health, New Hyde Park, NY, United States; ^2^ Sepsis Research Laboratory, The Feinstein Institutes for Medical Research, Manhasset, NY, United States

**Keywords:** cecal ligation and puncture, sepsis, innate immunity, organ dysfunction, T cell memory, CD4 T Cells, CD8 T cells, adaptive immunity

## Abstract

The role of T cell memory in sepsis is poorly understood. Recent work has demonstrated that mice exposed to frequent antigenic stimulation, in contrast to laboratory mice, better recapitulate the human T cell repertoire. This difference may profoundly alter responses to inflammatory insults. We induced isolated T cell memory by inoculating C57Bl/6 mice with an anti-CD3ϵ activating antibody, a process we term “immune education.” These mice were subjected to the cecal ligation and puncture (CLP) model of sepsis and responses were compared to those of isotype-treated controls. CLP-induced increases in 1) CD4 T cell production and serum levels of IFNγ, 2) CD8 T cell granzyme B levels, and 3) innate cell function were all more pronounced in educated mice than in control mice. Immune education increased CLP-induced liver injury and decreased survival. The differences in responses to CLP were not recapitulated in mice with either isolated CD4 or isolated CD8 T cell memory. Relative to controls, CLP in educated CD8^−/−^ mice (isolated CD4 memory) increased monocyte-derived dendritic cells. Combined CD4 and CD8 memory did not increase monocyte-derived dendritic cells; this combination recapitulated increases in neutrophil and inflammatory monocyte numbers in educated wild-type mice. Induction of T cell memory prior to CLP alters immune responses, organ function, and survival. Both CD4 and CD8 memory T cells play important and independent roles in this response. These findings have profound implications for the development of murine models of human inflammatory disorders such as infection and sepsis.

## Introduction

Sepsis is defined as life-threatening organ dysfunction caused by a dysregulated host response to infection (1). The disorder contributes to an estimated 5.3 million annual deaths worldwide (2). Implementation of evidence-based guidelines emphasizing early intervention with fluids, antibiotics, and supportive measures has improved outcome (3). However, a myriad of clinical trials have failed to identify any specific or effective therapeutic approach for this deadly disorder. Many of these trials were based on promising results from studies using animal models, most often cecal ligation and puncture (CLP) in rodents. These discrepancies have led to a re-evaluation of the model (4).

Recent work comparing laboratory mice to mice living in a “natural” environment has led to findings that may profoundly affect the CLP model. These studies demonstrated that mice living outside the laboratory setting are “immune educated.” These mice, which are exposed to multiple antigens and a more diverse microbiome, have higher numbers and proportions of memory T cells than mice kept in specific-pathogen free facilities (“immune naïve” or control mice) (5, 6). Study of immune educated mice identified a substantial and diverse population of circulating and solid-organ resident memory T cells (5, 6). The role of T cell memory in both clinical sepsis and CLP is poorly understood. In the experiments described here, we expand on the use of a novel, previously described method to induce a diverse repertoire of memory T cells without directly altering other aspects of the immune system (7). We then performed CLP on immune educated and control animals and compared responses. Our results demonstrate that the induction of T cell memory altered the CLP model in ways that may have important implications for translation of this model to human sepsis.

## Materials and Methods

### Mice

C57Bl/6J and B6.SJL (Ptprc^a^Pepc^b^/BoyJ) male mice were obtained from the Jackson Laboratory (Bar Harbor, ME) and maintained in the animal facility at the Feinstein Institutes for Medical Research. CD4^−/−^ (B6.129S2-CD4^tm1Mak^/J) and CD8^−/−^ (B6.129S2-CD8a^tm1Mak^/J) mice were obtained from the Jackson Laboratory and bred and maintained in our immunodeficient animal facility.

### Cecal Ligation and Puncture Procedure

CLP was performed with two 22-gauge punctures performed in series under isoflurane anesthesia as previously described (8). All animals that underwent CLP were approximately 16 weeks of age. Animals were resuscitated with 50 ml/kg sterile normal saline at the end of surgery through subcutaneous tissue injection. Fluid administration was repeated every 24 h for up to 48 h. Mice were euthanized at given time points after CLP with pentobarbital. Sham operation was identical to CLP but with omission of ligation or puncture.

### In Vivo Immunization

Ultra-LEAF Anti-mouse CD3ϵ Antibody (145-2C11, Biolegend, San Diego, CA, 50 µg in 200 µl sterile phosphate-buffered saline) and Ultra-LEAF isotype Armenian Hamster IgG control (HTK888) were administered to 11-week-old mice *via* retro-orbital venous sinus injection. Mice were monitored for adverse effects and rested for 35 days to allow for immune memory development and to avoid any continued activation effects. Details of the T cell response and long term phenotype and of the innate cell response following anti-CD3ϵ inoculation have been published separately (7). Briefly, anti-CD3ϵ treatment led to acute CD4 and CD8 T cell activation that dissipated by post-treatment day 5. By 35 days following treatment, no acute effector CD4 or CD8 T cells were detected. During this time period, numbers of central and effector memory CD4 T cells and effector memory CD8 T cells in the spleen, liver and lungs increased. This memory differentiation persisted through 135 days without any increase in anergy or T regulatory cells. No alteration was detected in the innate immune system at 35 days following anti-CD3ϵ treatment in unchallenged mice.

### Adoptive Transfer

35 days following immunization, C57Bl/6J mice were sacrificed, spleens were homogenized and T cells were isolated by negative selection using the pan-T cell isolation kit (Miltenyi, Bergisch Gladbach, Germany). Cells were then labeled (CD90, CD4, CD8, CD44, CD11a) and sorted. 1.5 million T cells (CD4, CD8 or both) were injected into untouched B6.SJL mice. A BD FACSAria was utilized for sorting. Controls were given saline vehicle. Seven days later, mice underwent CLP and were sacrificed for analysis at 24 h.

### Leukocyte Isolation

Spleens were obtained from sacrificed mice, were immediately weighed and subjected to digestion with DNAse (100 µg/ml) and Collagenase A (1 mg/ml) in complete media for 30 min at 37°C. Cells were resuspended following filtration through a 70-µm filter. Red blood cells were lysed, white cells were counted using a Countess II Automated Cell Counter (ThermoFisher, Waltham, MA) and spleen cells were analyzed. Liver leukocytes were obtained following homogenization of livers and filtration through a 70-µm filter. Leukocytes were isolated at the interface of a 35%/75% Percoll solution gradient (GE Healthcare, Chicago, IL). Peritoneal cells were obtained immediately following euthanasia through an abdominal incision. The peritoneum was washed with 5 ml of sterile saline and collected. All cell counts were performed using a Countess II Automated Cell Counter (ThermoFisher, Waltham, MA). A minimum of 2 × 10^6^ events were analyzed for each sample.

### Flow Cytometric Analysis

Once single-cell suspensions were obtained, cells were stained for flow cytometric analysis. Staining was performed with LIVE/DEAD fixable viability dye (Life Technologies) and the following antibodies: CD90.2, CD44, CD8a, CD4, CD62L, KLRG1, CD11a, Ly6C, CD11c, Ly6G, MHCII, IL1β, TNFα, IL2, IFNγ, and IL17a. Full antibody details are available in **Supplementary Table 1**. All flow cytometric analysis was performed on a BD LSR Fortessa 16-color cell analyzer and analyzed using FlowJo software version 10 (BD Bioscience, San Jose, CA). Gating strategies are listed in figure captions. Gating strategies are illustrated in **Supplementary Figure 1** for CD4 T cells, **Supplementary Figure 2** for CD8 T cells and **Supplementary Figure 3** for innate immune cells.

### Cytokine Production Assays

To assess cytokine production, once cells were in single cell suspension, they were placed with appropriate stimuli. For T cells, T cell receptor stimulation was performed through plate bound anti-CD3ϵ (5 µg/ml) and anti-CD28 (1.7 µg/ml) in solution. Cells were stimulated for 5 h in the presence of Brefeldin A (2 µg/ml). For innate cells, cells were stimulated with LPS (500 ng/ml) for 3 h in the presence of Brefeldin A. All stimulation assays were performed alongside a control without stimulation to assess for background production as previously described (9). For reactive oxygen species assessment, the CellRox Green Flow cytometry Assay Reagent (ThermoFisher, Waltham, MA) was used. This reagent reacts with reactive oxygen species and fluoresces in the FITC spectrum allowing for fluorescent detection of reactive oxygen species production. The change from baseline with LPS stimulation was used to determine ROS induction.

### ELISA Assays

ELISA assays were performed per manufacturer instructions. The MesoScale Discovery Multiplex (Rockville, MD) was used for initial analysis and findings were confirmed with BD OptEIA ELISA kits. Bilirubin Assay Kit (Sigma-Aldrich, St Louis, MO) and Alanine Aminotransferase 1 ELISA Kit (Biovision, Milpitas, CA) were used per manufacturer instructions.

### Real-Time Polymerase Chain Reaction

Samples were stored in RNALater solution (Invitrogen) until total RNA was isolated (RNAeasy Mini Kit; Qiagen, Hilden, Germany). Quantitative real-time PCR was performed using an Applied Biosystems 7900HT Fast Real-Time PCR system. Primers were obtained from Applied Biosystems: TaqMan Gene Expression Assays Mm00441421_m1 (SCL10a1a), Mm01267415_m1 (SCLO1a1), and Mm99999915_g1 (GAPDH). Relative amplification to GAPDH was calculated using the ΔΔCT method, which measures the change in the relative quantification of a control mRNA gene in an experimental sample and a control sample to the change in the relative quantification of a target mRNA gene of interest in those same samples.

### Statistical Analysis

Animal data were analyzed using Student’s two-way T test or using one-way analysis of variance where appropriate. Survival was calculated using the Spearman’s Log Rank Test (Prism 7.0; GraphPad, San Diego, CA). All data shown are representative of at least two independent experiments to ensure results. The authors declare that all data supporting the findings of this study are available within the paper and its **Supplementary Material**.

### Study Approval

All animal studies were approved by the Institutional Animal Care and Use Committee (IACUC #2017-039) and adhered to National Institutes of Health and Animal Research: Reporting of In Vivo Experiments (ARRIVE) guidelines.

## Results

### CLP Differentially Depletes Memory and Naïve T Cell Subsets in Uneducated (Control) Laboratory Mice

**Figure 1:**
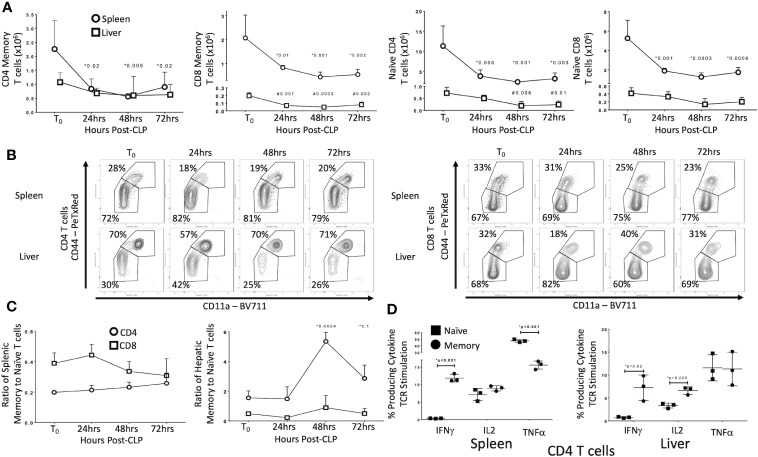
Effects of CLP on memory and naïve T cell subtypes in the spleen and liver. C57Bl/6 laboratory mice underwent CLP and were sacrificed at given timepoints. Data obtained using flow cytometry. Unoperated mice were used as T_0_ controls. Data as mean ± SEM, p<0.05 for spleen (*) or liver (#) compared to T_0_ by one-way ANOVA with Dunnett correction for multiple comparisons. Figures representative of at least two independent experiments. **(A)** Total CD4 and CD8 Memory and Naïve T cells as a function of time post-CLP. Spleen – circles, Liver – squares. Gating: FSC/SSC, singlets, Live, CD90/CD4, CD90/CD8, CD44/CD11a; N = 3–4/group. **(B)** Flow cytometric plots of CD4 (left) and CD8 T cells (right) by CD44/CD11a from the spleen (top) and liver (bottom) at T_0_, 24, 48, and 72 h with percentage of parent in gate. Gating: FSC/SSC, singlets, Live, CD90/CD4, CD90/CD8, CD44/CD11a; N = 3–4/group. **(C)** Ratio of splenic and hepatic memory to naïve T cells over time. Gating: FSC/SSC, singlets, Live, CD90/CD4, CD90/CD8, CD44/CD11a; N = 3–4/group. **(D)** Percentage of memory and naïve CD4 T cells producing cytokine after 5 h *ex vivo* stimulation with CD3/CD28. Gating: FSC/SSC, singlets, Live, CD90/CD4, CD90/CD8, CD44/CD11a, IFNγ, IL2, TNFα. N= 3/group. *p<0.05 for all studies by two-way Student’s T-test.

We first sought to identify the effects of CLP on T cell subsets in uneducated (control) laboratory mice. Data are detailed in **Figure 1**. In the spleen of unoperated (T0) mice, 72% of CD4 T cells and 67% of CD8 T cells were naïve (CD44^−^/CD11a^−^/CD62L^+^) or CD44^-^/CD11a^−^ effectors (CD44^-^/CD11a^−^/CD62L^−^). In the livers of T0 mice, CD44^+^/CD11a^+^ effector (CD44^+^/CD11a^+^/CD62L^−^) and central memory (CD44^+^/CD11a^+^/CD62L^+^) T cells constituted 70% of the CD4 T cells population (30% naïve), while 68% of CD8 T cells were naïve (32% memory). Populations in sham operated (SO) mice were similar (data not shown). By 24 h post-CLP the abundance of naïve and memory CD4 and CD8 T cells in the spleen decreased significantly; this decrease persisted at 48 and 72 h. In the liver, CLP did not affect the number of memory CD4 T cells or naïve CD8 T cells, but the number of naïve CD4 and memory CD8 T cells decreased (**Figures 1A, B**; full gating strategy detailed in **Supplementary Figures 1** and **2**). The ratio of memory-to-naïve CD4 and CD8 T cells in the spleen did not change, nor did the ratio of hepatic CD8 T cells (**Figure 1C**). The ratio of hepatic CD4 memory T cells to naïve T cells increased 3.5-fold by 48 h and remained elevated from T0 at 72 h **Figure 1C**). These findings indicate that, while the number of T cells either decreased or did not change in response to CLP, the relative contribution of CD4 memory T cells to the T cell population in the liver increased.

### CLP Differentially Alters T Cell Receptor (TCR)-mediated Activation of Memory and Naïve T Cells in Control Mice

A number of characteristics differentiate memory T cells from naïve T cells. Distinctions include early proliferation with antigen activation, early response to stimuli and resistance to apoptosis and overall enhancement of the immune response. Memory T cells are also frequently found in non-lymphoid organs and solid tissue, while naïve T cells in these locations are less common. These alterations associated with T cell memory suggest that these cells could play a more important role in CLP than do naïve T cells. As noted above, CLP increased the ratio of memory to naïve T cells in the liver. Therefore, we examined the effects of CLP on the percentage of naïve (CD44^-^/CD11a^−^) and memory (CD44^+^/CD11a^+^) T cells producing cytokines following isolation from either the spleen (lymphoid tissue) or liver (non-lymphoid tissue). Twenty-four hours after CLP in control mice, T cell receptor (TCR, CD3/CD28) stimulation of intracellular cytokine production in memory T cells differed from that observed in naïve T cells in both spleen and liver (**Figure 1D**). Responses were similar in CD4 and CD8 T cells (data not shown). In both the spleen and liver following CLP, IFNγ was produced by a higher percentage of memory CD4 and CD8 T cells compared to naïve T cells. In contrast, TNFα was produced primarily by naïve T cells in the spleen, while a significantly smaller proportion of memory T cells produced this cytokine. In the liver, a similar proportion of both memory and naïve T cells produced TNFα, while a greater fraction of memory T cells in the liver produced IL2. The percentages of memory and naïve T cells producing IL2 in the spleen did not differ (flow cytometric plots shown in **Supplementary Figures 1** and **2**). No difference in CD4 T regulatory cells (Tregs) was detected between control and educated mice 24 h following CLP (data not shown) although educated mice had a decreased percent of T regulatory cells prior to CLP (7).

### Serum IFNγ/IL12p70 Levels Following CLP Are Higher in Immune Educated Mice Than in Controls

**Figure 2:**
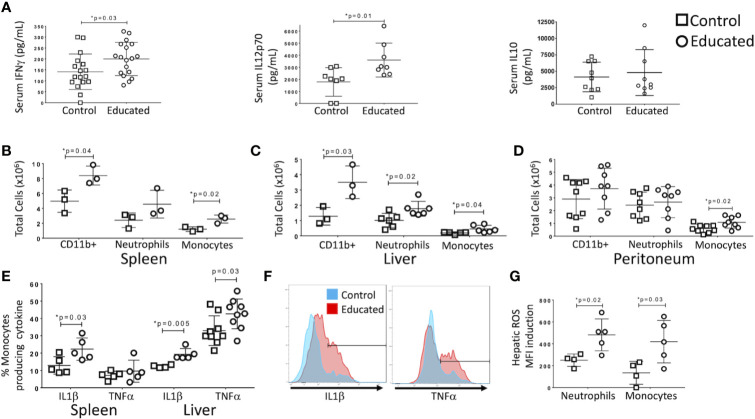
Effects of immune education on the innate immune response to CLP. C57Bl/6 mice were treated with anti-CD3ϵ (circles) or isotype control (squares) and subjected to CLP 35 days later. Mice were sacrificed at 24 h post-CLP. Gating for B-D: Leukocytes: FSC/SSC, singlets, Live, CD11c^-^/CD11b^+^; Neutrophils: FSC/SSC, singlets, Live, CD11b^+^/Ly6G^+^, ROS; also Ly6Cint; Inflammatory monocytes: FSC/SSC, singlets, Live, CD11c^-^/CD11b^+^, CD11b^+^Ly6G^-^, CD11b^+^/MHCII^-^, CD11b^+^/Ly6Chi, IL1α, TNFα, ROS. Shown in **Supplementary Figure 3**. Data as mean ± SEM, *p < 0.05 by two-way Student’s T test. Figures representative of at least two independent experiments. **(A)** Alteration in serum IFNγ, IL12p70, and IL10. N = 17–18/group for IFNγ, 8/group for IL12p70 and 9/group for IL10. **(B)** Number of innate immune cells obtained from the spleen. N = 3. **(C)** Number of innate immune cells obtained from the liver. N = 3. **(D)** Number of innate immune cells obtained from peritoneal wash. N = 8. **(E)** Percentage of inflammatory monocytes that responded to *ex vivo* LPS stimulation with IL1β or TNFα production. N = 5–8. Left: Spleen. Right: Liver. Cytokine production without LPS stimulation was minimal. Gating shown in **Supplementary Figure 3**. **(F)** Histograms comparing hepatic inflammatory monocyte production of IL1β (left) and TNFα (right) in response to *ex vivo* LPS stimulation. Red=Educated, Blue=Control. Representative of 5–8 animals. Comparison to unstimulated controls shown in **Supplementary Figure 3**. **(G)** Reactive Oxygen Species (ROS) induction by hepatic neutrophils and monocytes. ROS presence determined by increase in median fluorescence intensity (MFI) of CellRox reagent (see methods) following *ex vivo* stimulation with LPS. N = 5.

Following CLP, memory T cells were more likely to produce IFNγ than were naïve T cells (**Figure 1D**). CLP in control mice also increased the ratio of memory-to-naïve CD4 T cells in the liver. Given recent findings that the memory T cell proportion is significantly higher in wild and pet store mice compared to laboratory mice (5, 6), we examined the effect of a pre-surgical increase in the combined CD4 and CD8 memory T cell populations on the response to CLP. In previous studies, we used an anti-CD3ϵ activating antibody to induce broad T cell memory (7). Prior to CLP, serum cytokine levels in educated and control mice did not differ. To determine the effects of immune education on CLP-induced cytokine production, serum cytokine levels were measured 24 h after CLP. Levels of IFNγ and IL12p70, but not IL10, IL6 or TNFα, were significantly higher in educated mice than in controls (**Figure 2A**).

### Immune Education Enhances the Innate Immune Response to CLP

T cell-derived IFNγ induces production of IL12p70, a response that amplifies innate immune responses in a “feed-forward” loop (10, 11). Because immune education increased CLP-induced serum levels of both cytokines (**Figure 2A**), we examined the effects of immune education on CLP-induced activation of the innate immune system. The CLP-induced expansion of CD11b^+^ (myeloid lineage) cell populations in the spleen and liver was more profound in immune educated mice than in control mice (**Figures 2B, C**). No such difference was noted in the peritoneum (**Figure 2D**). Our previous study demonstrated that immune education increased CD4 and CD8 memory T cell proportions but did not alter the innate immune system (7). Thus, the presence of memory T cells increased the effects of CLP on the innate immune response. Further, the CLP-induced myeloid (CD11b^+^) cell expansion in educated mice resulted from an increase in Ly6Chi inflammatory monocytes (CD11b^+^/MHCII^-^/Ly6G^-^/Ly6Chi) in spleen, liver and peritoneum and of neutrophils (CD11b^+^/Ly6G^+^/Ly6C^+^/MHCII^-^) in the liver (**Figures 2B–D**), indicating T cell memory drives a neutrophilic and monocytic response to CLP in this system. Gating strategy and representative flow plots are shown in **Supplementary Figure 3**.

**Figure 3:**
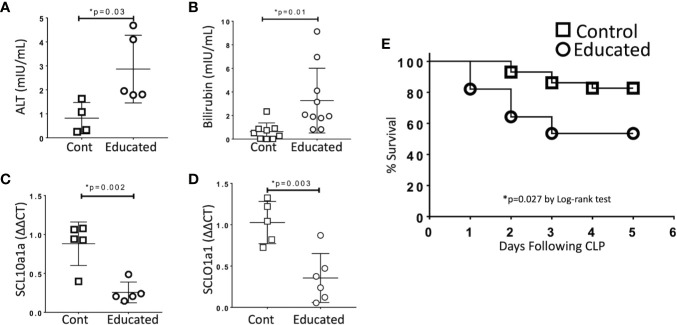
Effects of Immune Education on CLP-induced organ injury and CLP survival. C57Bl/6 mice were treated with anti-CD3ϵ (Educated – circles) or isotype control (Cont – squares) and were subjected to CLP 35 days later. Mice were sacrificed 24 h post-CLP, serum was obtained and RNA was extracted from liver. In a separate cohort, five-day survival from CLP was determined. Data as mean ± SEM, *p < 0.05 for all studies by two-way Student’s T test. Figures representative of at least two independent experiments. **(A)** Serum alanine aminotransferase (ALT) levels (N = 4–5/group). **(B)** Serum total bilirubin concentration (N = 9–10/group). **(C)** Relative hepatic abundance of mRNA encoding Sodium/Bile Acid Cotransporter (SCL10a1a). ΔΔCT values of each sample normalized to GAPDH to account for loading, mean value for abundance of control specimen arbitrarily set at unity. N= 5–6/group. **(D)** Relative hepatic abundance of mRNA encoding Organic Anion Transporter (SCLO1a1). ΔΔCT values of each sample normalized to GAPDH to account for loading, mean value for abundance of control specimen arbitrarily set at unity. N= 5–6/group. **(E)** Five-day Survival following CLP in educated (circles) and control (squares) mice. N = 28–29/group. p = 0.027 by Spearman’s Log-rank test.

LPS-stimulated intracellular cytokine production in inflammatory monocytes isolated following CLP in immune educated mice also differed from that observed in control animals. Effects specific to immune educated mice included 1) a higher percentage of splenic inflammatory monocytes that produced high levels of IL1β (**Figure 2E**), 2) a higher percentage of hepatic inflammatory monocytes that produced high levels of TNFα and IL1β (**Figure 2E**), and 3) higher overall production of IL1β (median fluorescence intensity) by hepatic monocytes (data not shown). Histograms of IL1β and TNFα production by hepatic monocytes are depicted in **Figure 2F**; unstimulated controls shown in **Supplementary Figure 3**. Further, both neutrophils and monocytes isolated from livers of immune educated mice following CLP produced more reactive oxygen species in response to LPS stimulation than did cells isolated from control mice (**Figure 2G**). In aggregate, these findings indicate that the induction of T cell memory significantly enhanced the CLP-induced innate immune response. The augmented response was particularly evident in the liver, suggesting that immune education effected lymphoid organs and solid organs differently.

### Immune Education Enhances CLP-induced Hepatic Dysfunction

Organ dysfunction is the defining characteristic of sepsis (1). To examine the effects of immune education on hepatic dysfunction, we compared responses in control and educated mice at 24 h following CLP. Specifically, we measured the effects of immune education on the hepatic response to CLP using standard measures of liver injury (transaminases, bilirubin). In addition, based on results from previous studies, we examined the effects of immune education on the CLP-induced reduction in the expression of the genes encoding the sodium/bile acid cotransporter (SCL10a1a) and the organic anion transporter protein 1 (SCLO1a1) in the liver (12). Data are detailed in **Figure 3**. CLP-induced increases in serum alanine aminotransferase (ALT, **Figure 3A**) and bilirubin (**Figure 3B**) and decreases in the abundance of mRNA encoding SCL10a1a (**Figure 3C**) and SCLO1a1 (**Figure 3D**) were more profound in immune educated mice.

### Immune Education Decreases Survival Following CLP

Cohorts of educated and control mice were subjected to CLP and were followed for five days. Survival in educated mice was significantly lower than that observed in controls (**Figure 3E**).

### CLP-Induced Changes in the Abundance of Different T Cell Subtypes Are Altered by Immune Education

**Figure 4:**
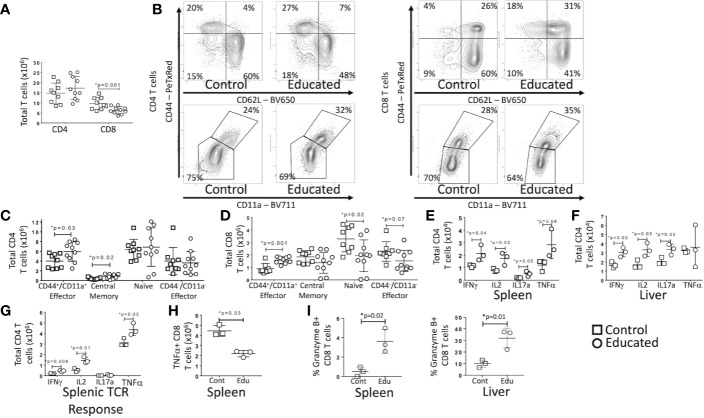
Effects of Immune Education on T cell response to CLP. C57Bl/6 mice were treated with anti- CD3ϵ (circles) or isotype control (squares) and 35 days later were subjected to CLP. 24 h post-CLP, mice were sacrificed and specific assays were performed. Gating: FSC/SSC, singlets, Live, CD90/CD4, CD90/CD8, CD44/CD62L for subset analysis, IFNγ, IL2, IL17a, TNFα, granzyme B for cytokine studies and granzyme B expression. Data as mean ± SEM, *p < 0.05 by Student’s two-way T test. Figures representative of at least two independent experiments. **(A)** Total number of splenic CD4 and CD8 T cells. N = 9–11 per group. **(B)** Flow cytometric plots of CD4 (left) and CD8 T cells (right) by CD44/CD62L (top) and CD44/CD11a (bottom) from the spleen 24 h following CLP in control and educated mice with percentage of parent in gate. Gating: FSC/SSC, singlets, Live, CD90/CD4, CD90/CD8, CD44/CD62L or CD44/CD11a; N = 3–5/group. **(C)** Total number of splenic CD4 T cell subsets. N = 9–11 per group. **(D)** Total number of splenic CD8 T cell subsets. N = 9–11 per group. **(E)** Total splenic T cells spontaneously producing IFNγ, IL2, IL17a and TNFα. Cells treated with Brefeldin A for 5 h prior to intracellular cytokine staining. N = 3. **(F)** Total hepatic T cells spontaneously producing IFNγ, IL2, IL17a and TNFα. Cells treated with Brefeldin A for 5 h prior to intracellular cytokine staining. N = 3. **(G)** Total splenic CD4 T cells producing IFNγ, IL2, IL17a and TNFα in response to TCR stimulation. Cells treated with Brefeldin A for 5 h prior to intracellular cytokine staining. N = 3. **(H)** Total splenic CD8 T cells producing TNFα in response to TCR stimulation. Cells treated with Brefeldin A for 5 h prior to intracellular cytokine staining. N = 3. **(I)** Percent CD8 T cells expressing granzyme B in the spleen and liver. Left – Spleen, Right - Liver N = 3.

Both CLP and induction of T cell memory change the relative numbers of specific T cell subtypes (13, 14). We have previously demonstrated that immune education expanded the central and effector memory CD4 T cell population and the effector memory CD8 T cell in multiple solid organs (7). We therefore examined CLP-induced changes in T cell sub-populations in educated and control mice. Data are depicted in **Figure 4**.

At 24 h post-CLP, the total number of CD4 T cells in the spleen of educated mice did not differ from the number observed in controls. The number of CD8 T cells was, however, significantly lower in educated mice than in controls (**Figure 4A**). Despite the absence of a difference in total CD4 T cells, there were significant differences in CD4 T cell subsets post-CLP in educated and control mice. Following CLP, both percentage and numbers of splenic central memory (CD44^+^/CD62L^+^) and CD44^+^/CD11a^+^ effector (CD44^+^/CD62L^−^) CD4 T cells (**Figures 4B, C**) were higher in educated mice than in control mice; CD11a expression was also increased. The numbers of splenic naïve (CD44^-^/CD62L^+^) and CD44^-^/CD11a^−^ effector (CD44^-^/CD62L^−^) CD4 T cells were unchanged. The number of naïve (CD44^-^/CD62L^+^) CD8 T cells in the spleen was lower in educated mice than in control mice, while the percentage and number of CD44^+^/CD11a^+^ effector CD8 T cells was greater in educated mice than in control mice (**Figures 4B, D**). These differences explained the global differences in numbers of CD8 T cells depicted in **Figure 4A**. Consistent with the expansion of the CD4 T cell memory population, the effects of CLP on the number of CD4 T cells spontaneously producing IFNγ, IL2 and IL17a during CLP in both the spleen (**Figure 4E**) and the liver (**Figure 4F**) was greater in educated mice than in controls (flow cytometric plots of intracellular cytokine staining shown in **Supplementary Figures 1** and **2**). In addition, the number of CD4 T cells in the spleen 24 h following CLP that responded to TCR stimulation by producing IFNγ, IL2 and TNFα was greater in immune educated mice than in control mice (**Figure 4G**). However, the number of splenic CD8 T cells that, when subjected to TCR stimulation 24 h following CLP, produced TNFα was lower in educated mice than in control mice (**Figure 4H**). We did not observe any other significant changes in the CD8 T cell cytokine response.

CD8 T cells also have cytolytic capacity that is enhanced by T cell memory. Granzyme B is both a marker of CD8 T cell memory and an inducer of cytolytic apoptosis in target cells during immune synapse formation. Granzyme B expression was low in splenic CD8 T cells 24 h following CLP in both control and immune educated mice, although there was a significant increase in the percentage of granzyme B expressing cells in educated mice. A significantly greater proportion of hepatic CD8 T cells expressed granzyme B; almost 35% of hepatic CD8 T cells in educated mice expressed granzyme B while approximately 10% of hepatic CD8 T cells expressed granzyme B in control mice (**Figure 4I**, flow cytometric plots shown in **Supplementary Figure 2**). Almost all granzyme B expressing CD8 T cells had a memory phenotype (CD44^+^/CD11a^+^, data not shown) indicating that immune education increased the cytolytic capacity of hepatic memory CD8 T cells.

### CLP-Induced Increases in Innate Immune Responses in Educated Mice Depend on Both CD4 and CD8 Memory As Well As Monocyte-derived Dendritic Cells

Immune education induced both CD4 and CD8 T cell memory. Further, both CD4 and CD8 T cells contribute to the CLP response (15, 16). To identify the contribution of immune education of CD4 and CD8 T cell education to CLP-induced activation of innate immunity, we used CD4^−/−^ (ie, CD8 T cells only) and CD8^−/−^ (ie, CD4 T cells only) mice. CLP was performed 35 days after immune education and mice were sacrificed 24 h later. Mice were compared to isotype treated CD4^−/−^ and CD8^−/−^ mice.

**Figure 5:**
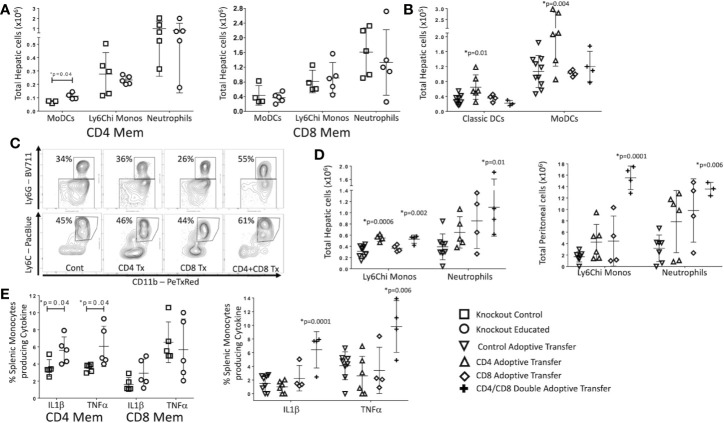
Effects of isolated and combined CD4 and CD8 memory on CLP-induced changes in the innate immune system. CLP was performed on educated (circles) and control (squares) CD4^−/−^ (CD8 T cells only) and CD8^−/−^ (CD4 T cells only) mice. In a separate cohort, flow-cytometry assisted sorted CD4 memory T cells, CD8 memory T cells or both were transferred into congenic B6.SJL mice. Seven days later, these mice and untreated control mice underwent CLP. In all groups, liver and spleen were harvested 24 h following CLP and peritoneal cavity washings were obtained. Cell samples were analyzed using flow cytometry. Gating: Monocyte-derived Dendritic Cells (MoDCs): FSC/SSC, singlets, Live, CD11c^-^/CD11b^+^, CD11b^+^/Ly6G^-^, CD11b^+^/MHCII^+^; Inflammatory monocytes: FSC/SSC, singlets, Live, CD11c^-^/CD11b^+^, CD11b^+^Ly6G^-^, CD11b^+^MHCII^-^, CD11b^+^/Ly6Chi; Neutrophils: FSC/SSC, singlets, Live, CD11c^-^/CD11b^+^, CD11b^+^Ly6G^+^, also Ly6Cint; Classical dendritic cells: FSC/SSC, singlets, Live, CD11c^+^/CD11b^-^, CD11c^+^/MHCII^+^. N = 4–5/group for knockout mice, N = 4–9 for adoptive transfer experiments. Data as mean ± SEM, *p < 0.05 for all studies by two-way T test used in experiments involving knockout mice and by one-way ANOVA with Dunnett correction for multiple comparisons in adoptive transfer experiments. Figures representative of at least two independent experiments. Experimental data pooled for adoptive transfer. **(A)** Total moDCs, Ly6Chi monocytes and neutrophils in the liver of CD8^−/−^ mice (CD4 memory T cells only) or CD4^−/−^ mice (CD8 memory T cells only). Control (squares) and Educated (circles) mice. **(B)** Total classical DCs and moDCs in the liver following adoptive transfer and CLP. Controls (squares), CD4 memory T cells (inverted triangles), CD8 memory T cells (diamonds), both CD4 and CD8 memory T cells (crosses). **(C)** Flow cytometric plots of neutrophils (CD11b+/Ly6G+, top) and inflammatory monocyotes (CD11b^+^/Ly6G^-^/MHCII^-^/Ly6Chi, bottom) from the liver 24 h following CLP in mice following adoptive transfer of CD4 T cells, CD8 T cells, both CD4 and CD8 T cells or sham injection. Neutrophils: FSC/SSC, singlets, Live, CD11c^-^/CD11b^+^, CD11b^+^Ly6G^+^, also Ly6Cint; Inflammatory monocytes: FSC/SSC, singlets, Live, CD11c^-^/CD11b^+^, CD11b^+^Ly6G^-^, CD11b^+^MHCII^-^, CD11b^+^/Ly6Chi; N = 3–5/group. **(D)** Hepatic and peritoneal Ly6Chi monocytes and neutrophils following adoptive transfer and CLP. Controls (inverted triangles), CD4 memory T cells (triangles), CD8 memory T cells (diamonds), or both CD4 and CD8 memory T cells (crosses). **(E)** Percent of splenic monocytes producing cytokines following Brefeldin A treatment and *ex vivo* LPS stimulation for 3 h Left: Effects of CLP on control (squares) and educated (circles) CD8^−/−^ mice (CD4 memory T cells only) or CD4^−/−^ mice (CD8 memory T cells only). Right: Effects of CLP on controls (inverted triangles), mice with adoptive transfer of CD4 memory T cells (triangles), mice with adoptive transfer of CD8 memory T cells (diamonds), and mice with adoptive transfer of both CD4 and CD8 memory T cells (crosses).

In mice with only CD8 T cells (CD4^−/−^), immune education did not alter the innate immune response to CLP. However, in mice with only CD4 T cells (CD8^−/−^), splenic neutrophil counts 24 h post-CLP were greater in educated mice than in control mice (**Supplementary Figure 4**). In contrast to findings in wild type mice (**Figures 2B–D**), immune education in either CD4^−/−^ or CD8^−/−^ mice failed to affect CLP-induced changes in hepatic or peritoneal inflammatory monocyte or neutrophil counts (**Figure 5A**). However, CLP-induced increase in hepatic and peritoneal monocyte derived dendritic cells (moDCs, CD11b^+^/CD11c^-^Ly6G^-^, MHCII^+^, gating strategy shown in **Supplementary Figure 3**) were more profound in immune educated CD8^−/−^ than in control CD8^−/−^ (**Figure 5A**). The CLP-induced increase in moDCs in wild-type mice or CD4^−/−^ (ie CD8^+^) mice was not altered by immune education. These findings suggest that CD4 memory T cells contribute to moDC development, while CD8 memory T cells may negate this effect.

To determine if the effects of immune education on CLP-induced changes in innate immunity required both CD4 and CD8 T cell memory, we isolated CD4 and CD8 memory (CD44^+^CD11a^+^) T cells from educated wild type C57Bl/6 mice. Post-sort purity was greater than 90% memory T cells (CD44^+^/CD11a^+^) for both CD4 and CD8 T cell populations (**Supplementary Figure 5**). Memory CD4 and/or CD8 T cells (1.5 x 10^6^ cells) were transferred to congenic B6.SJL mice. Wild type C57Bl/6 mice express CD45.2 on all hematopoietic cells while B6.SJL mice express the isoform CD45.1; therefore, transferred T cells can be distinguished from endogenous T cells. These animals rested for 7 days and were then subjected to CLP. Following CLP, transferred (CD45.2^+^/CD45.1^-^) CD4 T cells constituted approximately 0.2% of total CD4 T cells, while transferred CD8 T cells constituted approximately 0.24% of total CD8 T cells (**Supplementary Figure 6**). CD45.2^+^/CD45.1^-^ cells were not detected in control mice, CD4^+^/CD45.2^+^/CD45.1^-^ were not detected in mice injected with CD8 T cells and CD8^+^/CD45.2^+^/CD45.1^-^ were not identified in B6.SJL mice injected with CD4 T cells. Transferred cells maintained memory T cell phenotypes as indicated by CD44/CD62L/CD11a expression (**Supplementary Figure 6**) The effects of memory CD4, memory CD8 or double adoptive transfer on the innate immune response to CLP were compared to sham injected mice.

Results mirrored findings in knockout mice. Specifically, 24 h following CLP, the livers of memory CD4 T cell injected mice contained more moDCs and classical (CD11c^+^/MHC^+^, gating strategy shown in **Supplementary Figure 3**) dendritic cells than the livers of sham injected mice (**Figure 5B**). Adoptive transfer of memory CD8 T cells alone did not affect the response to CLP in hepatic moDCs (**Figure 5B**). Importantly, the effects of CLP on hepatic moDCs in mice subjected to double adoptive transfer (both CD4 and CD8 memory T cells) did not differ from sham injected control mice (**Figure 5B**). These findings indicate that memory CD4 T cells induced moDCs but this change was blocked or reversed through an interaction with CD8 memory T cells with the effects of CD4 memory T cells (**Figure 5B**). At 24 h post-CLP, the number of inflammatory monocytes in the liver was higher in mice that received either memory CD4 T cells or that received double adoptive transfer. The percentage of Ly6Chi monocytes of total monocytes was only increased in mice that received double adoptive transfer (**Figures 5C, D**). In contrast to CLP-mediated effects on hepatic moDC numbers, mice receiving double adoptive transfer responded to CLP with an increase in the percentage of hepatic neutrophils of total CD11b cells and an increase in the number of hepatic and peritoneal neutrophils (**Figures 5C, D**). When combined with data regarding effects of immune education on CLP-induced responses in CD4^−/−^ or CD8^−/−^ mice, these findings indicate that the effects of immune education on the CLP-induced changes in neutrophils and monocytes requires both CD4 and CD8 T cell memory (**Figures 5B–D**).

Finally, we investigated the contribution of CD4 and/or CD8 memory T cells to CLP-induced changes in innate immune cell function following CLP. Twenty-four hours following CLP, splenic cells were obtained from educated or control CD4^−/−^ or CD8^−/−^ mice and stimulated ex vivo with LPS. Following CLP, the percentage of monocytes producing of IL1β and TNFα was greater in educated CD8^−/−^ mice than in control knockout mice (**Figure 5E**), suggesting memory CD4 T cells were responsible for the increase in cytokine production. However, a similar change in the *ex vivo* response to LPS was noted only in mice subjected to double adoptive transfer. The response to stimulation in educated mice did not differ from that observed when control mice were subjected to CLP after adoptive transfer of either CD4 or CD8 memory T cells alone (**Figure 5E**). These findings suggest a role for naïve CD8 T cells; while CD8^−/−^ mice lack both naïve and memory CD8 T cells, naïve CD8 T cells (but not memory T cells) are present in mice subjected to CD4 adoptive transfer.

## Discussion

The experiments detailed in this report demonstrate that T cell memory altered several key aspects of the response to CLP. Specifically, T cell memory affected 1) innate and adaptive immune responses, 2) organ dysfunction, and 3) mortality. The implications are profound. All adult humans have a significant and unique repertoire of memory T cells that arise from a lifetime of antigenic exposures. This repertoire is not, however, replicated in laboratory mice because these animals are maintained in an isolated, pathogen-free environment and exposure to antigenic stimulation is limited (6) Therefore, the absence of memory T cells represents a crucial difference between CLP, the most commonly-used animal model of sepsis, and human sepsis itself.

CLP and other animal models have provided important information regarding sepsis pathobiology. These models have not, however, identified therapeutic approaches that have advanced the treatment of sepsis. Several potential reasons for these difficulties arise when the recent re-definition of sepsis as “life-threatening organ dysfunction caused by a dysregulated host response to infection” is considered (1). Adult humans have a significant memory T cell compartment that laboratory mice lack (6). Our work demonstrates that the introduction of immune education alters CLP-induced changes in both innate and adaptive immunity, suggesting that memory T cells contribute to “immune dysregulation.” Similarly, immune education increased hepatic dysfunction, suggesting that memory T cells play a key role in the development of organ dysfunction. Importantly, nearly all theoretical constructs of sepsis pathobiology are based on immune dysregulation/dysfunction – and most therapeutic approaches, many arising from experiments in laboratory mice, have focused on correcting these abnormalities. Thus, the lack of a robust memory T cell compartment in previous murine studies constitutes a critically important gap in our ability to translate findings from mice to human sepsis. Further, CD4 and CD8 T cell memory interaction is necessary to recapitulate findings in wild-type mice, indicating that involvement of these cells in the immune response independently could modulate human responses to sepsis and represent unique targets for future studies. Our results indicate that combined CD4 and CD8 T cell memory contribute independently to changes in this model. Combined T cell memory is rarely accounted for in animal models of human disease, nor is nonspecific T cell memory.

These studies have limitations. We induced isolated T cell memory. This is a model that allowed us to manipulate the T cell response specifically, but may not fully reflect the complexity of the human system as other parts of the immune system are likely changed by immunologic experiences. A recent report by Huggins, et al., noted changes in the CLP model in laboratory mice co-housed with pet store mice (17). The results were modulated by Toll-like receptor (TLR) – 4 and were associated with diversification of the microbiome, consistent with findings reported by others under different experimental conditions (18–22). As in the findings reported here, CLP-induced mortality and serum cytokine levels increased in co-housed mice, as did IFNγ production by T cells in response to Listeria infection. Thus, the findings in these two studies complement each other. The Huggins et al. model may more closely replicate the manner in which the memory T cell compartment develops in nature, allowing for identification of how interactions between the innate and adaptive immune systems develop. In contrast, our approach focused solely on the T cell memory response.

Another method utilized to induce increased T cell memory has been inoculation of mice with serial infections with *Listeria monocytogenes* and lymphocytic choriomeningitis virus (LCMV), both of which induce a T cell mediated immune response. Utilization of this model has been used to demonstrate increased T cell loss mediated by upregulation of 2B4 coinhibitory receptor during CLP challenge. While this model may mimic bacterial challenge following specific viral or bacterial infection, the T cell clonal memory developed in response to Listeria and LCMV are very specific with well-defined, finite epitope specificity. The anti-CD3 model activates a far more diverse repertoire of T cell clones, likely causing variation in the response to CLP (23).

We also examined T cell memory formed through uniform induction. While the anti-CD3 T cell memory model likely leads to development of diverse T cell memory without alteration of the innate immune system, the method of T cell activation through dendritic cell synapse formation through Fc receptor binding is artificial and may bias the formation of T cell memory in a specific immunologic direction. Therefore, the true human physiologic T cell response may be far more varied. Variation in human genetics or the environment at the time of memory induction may lead to alteration in the T cell response. Further study of other aspects of an experienced immune system are needed, but mechanistic understandings from these models will be extremely difficult due to the inherent complexity and variability of a mouse or human exposed to a lifetime of antigenic exposure. Similarly, to begin to translate these findings to human disease, the diversity of the T cell response during sepsis must be examined. The variability of the human T cell repertoire is profound even in normal healthy humans, but patterns in the T cell response to sepsis may occur given specific infections and sepsis phenotypes. This work supports pursuing examination of these responses during sepsis to begin to identify sepsis phenotype subsets that may portend different responses to treatments or outcomes.

The contribution of immune education on the liver requires additional comment. We chose to examine hepatic changes because we have also demonstrated that immune education alters the immune cell repertoire in the liver. Our work revealed that T cell memory exacerbated CLP-induced hepatic dysfunction. These findings suggest a link between immune responses and organ dysfunction that is in part mediated by T cell memory. Future studies will examine 1) direct effects of memory T cells themselves, 2) the importance of antigen-specific and/or nonspecific, antigen independent T cell activity, 3) the role of the varied T cell subsets, and 4) the contribution of memory T cell-mediated effects on other immune cells, such as neutrophils or inflammatory monocytes. Future studies will also examine effects of immune education on CLP-induced dysfunction in other organ systems.

Perhaps most importantly, mortality is the outcome variable most often assessed in both human clinical trials and following CLP. Indeed, translation from bench to bedside is usually based on the ability of a therapeutic approach to improve survival in an animal model. Our studies revealed that induction of a broad and robust memory T cell compartment prior to CLP significantly decreased survival. These finding suggest that sepsis therapies developed in lab mice, that is, in the absence of a robust memory T cell repertoire, are likely to fail in humans whose immune systems are rich in memory T cells. Clinical trials in humans confirm the validity of this statement as very few have successfully translated into successful treatment.

In summary, we have shown that the presence of memory T cells alters the immune response, organ dysfunction and survival following CLP. These three are the defining characteristics identified in the current definitions of sepsis (1). We believe that the presence of a robust memory T cell repertoire adds an element of construct validity to CLP, providing a model that more closely resembles adult human sepsis. Future studies into sepsis and, indeed, all inflammatory disorders should consider the contribution of T cell memory.

## Data Availability Statement

The original contributions presented in the study are included in the article/**Supplementary Material**; further inquiries can be directed to the corresponding author.

## Ethics Statement

The animal study was reviewed and approved by Institutional Animal Care and Use Committee of Northwell Health.

## Author Contributions

MT designed the research study, conducted the experiments, acquired and analyzed all data, and wrote the manuscript. TF, AK, and MA assisted in all animal experiments, performed procedures, assisted in experimentation, and reviewed the manuscript. CD assisted in study design, data analysis, assisted in writing the manuscript, and supervised all aspects of the research. All authors contributed to the article and approved the submitted version.

## Funding

MT received funding from the NIH NIGMS K08GM132794 and from the Thrasher Research Fund Early Career Award 14734. CD received funding from the NIH NIGMS R01GM121102.

## Conflict of Interest

CD is a consultant for Enlivex Therapeutics Inc, Jerusalem, Israel.

The remaining authors declare that the research was conducted in the absence of any commercial or financial relationships that could be construed as a potential conflict of interest.
