# Supplementary material for: CD4 and CD8 T Cell Memory Interactions Alter Innate Immunity and Organ Injury in the CLP Sepsis Model
Source: Front Immunol. 2020 Nov 20;11:563402. doi: 10.3389/fimmu.2020.563402 (PMC7715000; doi:10.3389/fimmu.2020.563402)
Supplement: Supplementary file 1 [file DataSheet_1.pdf]

Target	Clone	Cat. Number	Company	Fluorophore	Host Species	Target Species
CD90.2 (Thy1.2)	53-2.1	140318	Biolegend	BV605	Rat	Mouse
CD44	IM7	61-0441082	eBioscience	PE-eFluor610	Rat	Mouse/Human
CD8a	53-6.7	100730	Biolegend	AF700	Rat	Mouse
CD4	RM4-5	563727	BD Horizon	BV786	Rat	Mouse
CD62L	MEL-14	564108	BD Bioscience	BV650	Rat	Mouse
CD11a	M17/a	740676	BD Bioscience	BV711	Rat	Mouse
Ly6C	HK1.4	128013	Biolegend	Pac Blue	Rat	Mouse
Ly6C	HK1.4	128024	Biolegend	AF700	Rat	Mouse
CD11c	HL3	564079	BD Bioscience	BV650	Hamster	Mouse
Ly6G	1A8	127643	Biolegend	BV711	Rat	Mouse
CD11b	M1/70	101256	Biolegend	PE/Dazzle594	Rat	Mouse/Human
MHCII (I-A/I-E)	M5/114.15.2	17-5321-81	eBioscience	APC	Rat	Mouse
TNFa	MP6-XT22	506318	Biolegend	PacBlue	Rat	Mouse
IL17a	TC11-18H10.1	506920	Biolegend	PerCP/Cy5.5	Rat	Mouse
IFN gamma	XMG1.2	25-7311-82	eBioscience	PE/Cy7	Rat	Mouse
IL1 beta	NJTEN3	47-7114-82	eBioscience	APC-eFluor700	Rat	Mouse
IL2	JES6-5H4	503814	Biolegend	AF647	Rat	Mouse
Granzyme B	QA16A02	372214	Biolegend	PE/Cy7	Mouse	Human/Mouse
In Vivo						
Ultra-LEAF Purified anti-Mouse CD3e	145-2C11	100359	Biolegend		Hamster	Mouse
Ultra-LEAF Purified Armenian Hamster IgG Isotype Ctrl	HTK888	400959	Biolegend		Hamster	Mouse

Supplemental Table 1. Antibodies used in this work.

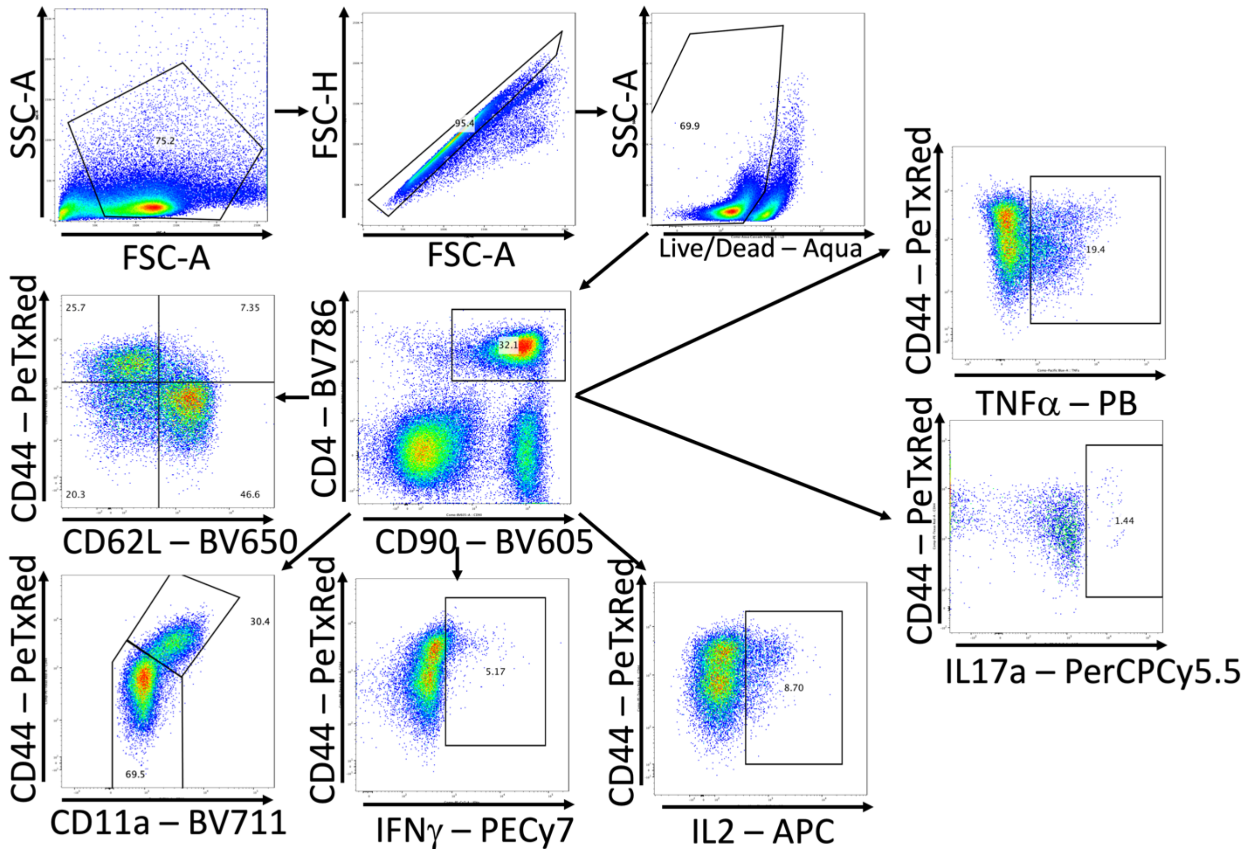

Supplemental Figure 1. Gating Strategy for CD4 T cells. Splenic T cell populations shown from educated mouse 24h following CLP without TCR stimulation for all except cytokine staining, which is shown following TCR stimulation.

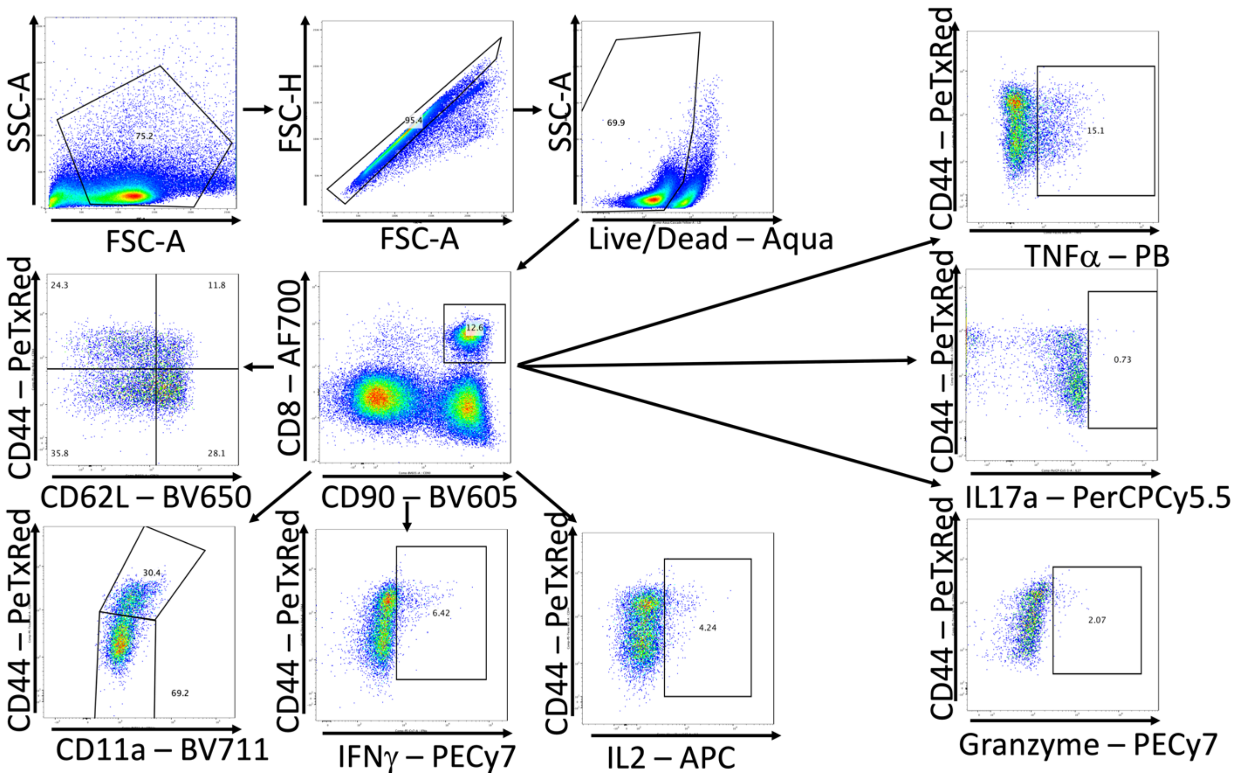

Supplemental Figure 2. Gating Strategy for CD8 T cells. Splenic T cell populations shown from educated mouse 24h following CLP without TCR stimulation for all except cytokine staining, which is shown following TCR stimulation.

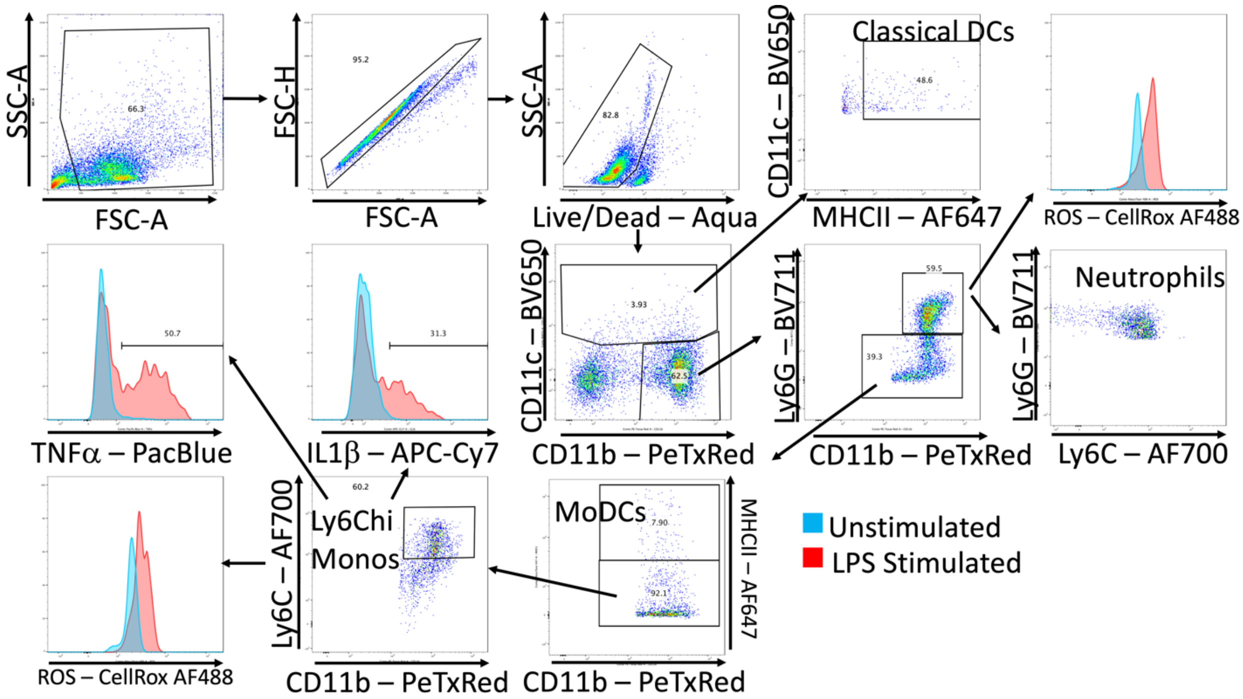

Supplemental Figure 3. Gating Strategy for Innate Immune cells. Hepatic innate immune populations shown from educated mouse 24h following CLP. For cytokine flow cytometric diagrams, red represents LPS stimulated sample and blue is control unstimulated sample from the same mouse.

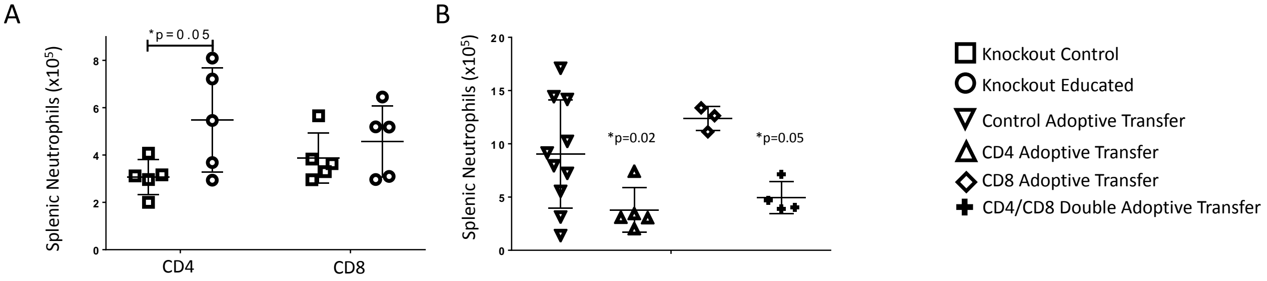

Supplemental Figure 4. Effects of isolated and combined CD4 and CD8 memory on CLP-induced changes in the innate immune system. CLP was performed on educated (circles) and control (squares) $CD4^{-/-}$ (CD8 T cells only) and $CD8^{-/-}$ (CD4 T cells only) mice. In a separate cohort, flow-cytometry assisted sorted CD4 memory T cells, CD8 memory T cells or both were transferred into congenic B6.SJL mice. Seven days later, these mice and untreated control mice underwent CLP. In all groups, liver and spleen were harvested 24h following CLP and peritoneal cavity washings were obtained. Cell samples were analyzed using flow cytometry. Gating: moDCs: FSC/SSC, singlets, Live, $CD11c^-$/$CD11b^+$, $CD11b^+$/$Ly6G^-$, $CD11b^+$/$MHCII^+$; Inflammatory monocytes: FSC/SSC, singlets, Live, $CD11c^-$/$CD11b^+$, $CD11b^+Ly6G^-$, $CD11b^+MHCII^-$, $CD11b^+$/Ly6Chi; Neutrophils: FSC/SSC, singlets, Live, $CD11c^-$/$CD11b^+$, $CD11b^+$Ly6Cint, Classical DCs: FSC/SSC, singlets, Live, $CD11c^+$/$CD11b^-$, $CD11c^+$/$MHCII^+$. N=4-5/group for knockout mice, N=4-9 for adoptive transfer experiments. Data as mean ± SEM, *$p<0.05$ by two-way T test for experiments involving knockout mice and by 2-way ANOVA with Dunnett correction for multiple comparisons for adoptive transfer experiments.

a. Total splenic neutrophils in $CD8^{-/-}$ mice (CD4 memory T cells only) or $CD4^{-/-}$ mice (CD8 memory T cells only). Control (squares) and Educated (circles) mice.

b. Total splenic neutrophils following adoptive transfer of CD4, CD8 or both CD4 and CD8 memory T cells or sham control 24h following CLP 7 days following transfer. Controls (squares), CD4 memory T cells (triangles), CD8 memory T cells (diamonds), both CD4 and CD8 memory T cells (circles).

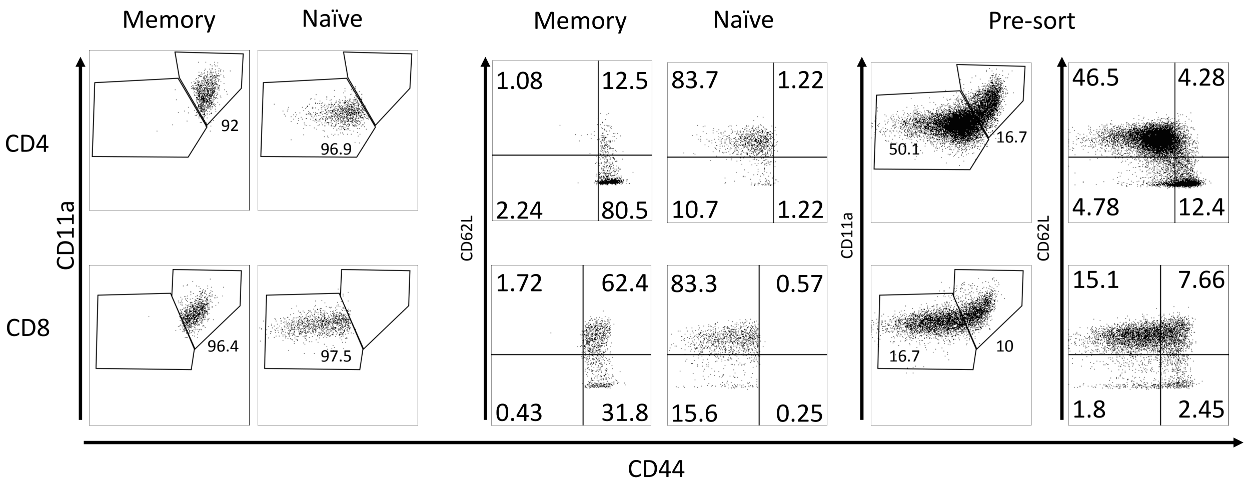

Supplemental Figure 5. Fluorescence assisted cell sorting diagram of CD4 or CD8 memory T cells (CD44^{+}/CD11a^{+}), with post-sort purity. CD62L/CD44 purity is also shown along with pre-sort diagrams.

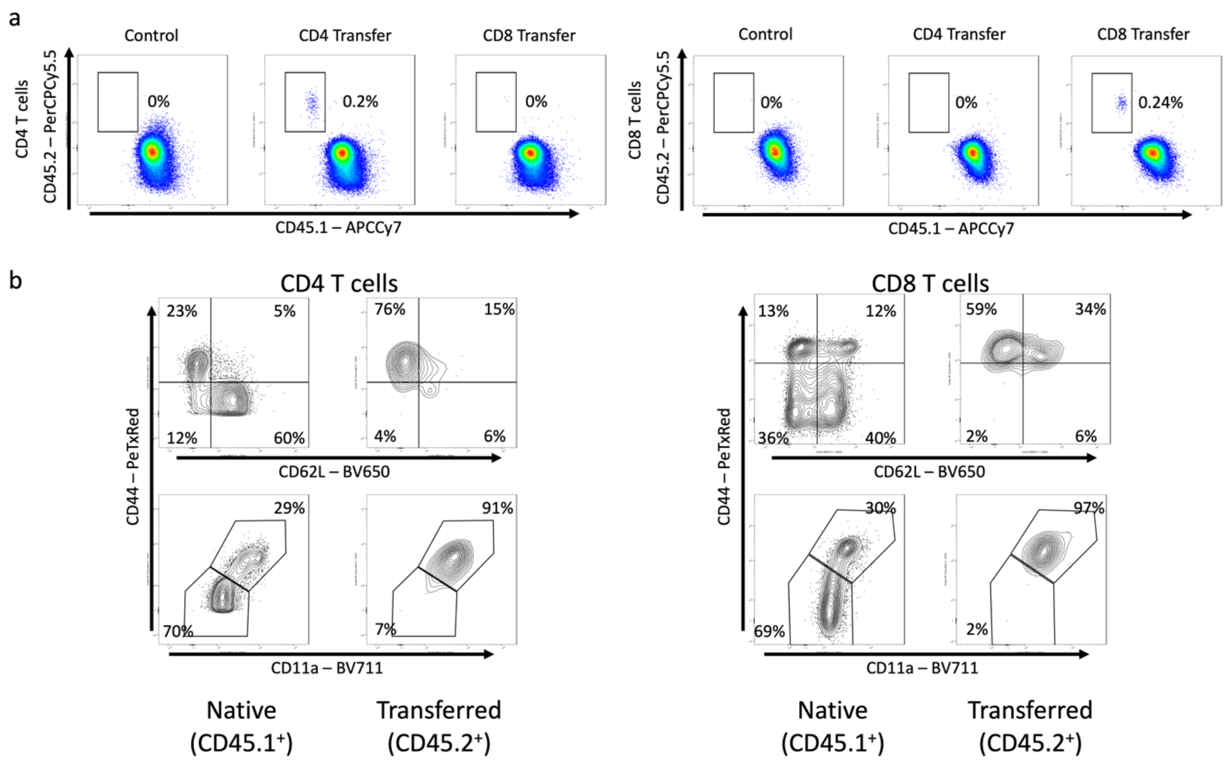

Supplemental Figure 6. Adoptive Transfer Efficiency. Flow-cytometry assisted sorted CD4 memory T cells or CD8 memory T cells from C57Bl/6 (CD45.2$^+$) mice were transferred into congenic B6.SJL (CD45.1$^+$) mice. Seven days later, these mice and untreated control mice underwent CLP. Spleens were harvested 24 hrs. following CLP. Splenic T cells were analyzed for CD45.1 expression (native) and CD45.2 expression (transferred), and for markers of T cell memory (CD44/CD62L/CD11a). Gating: FSC/SSC, singlets, Live, CD90/CD4, CD90/CD8, CD45.1/CD45.2, CD44/CD62L or CD44/CD11a. N=3-5/group

a. Flow cytometric plots of CD4 T cells (left) and CD8 T cells (right) by CD45.1/CD45.2. Gate shows transferred T cells, number represents percentage of parent.

b. Flow cytometric plots of native (CD45.1$^+$) and transferred (CD45.2$^+$) CD4 (left) and CD8 (right) T cells by CD44/CD62L (top) and CD44/CD11a (bottom). Numbers represent percentage of parent in gate.
